# Beyond Dopamine: GABA, Glutamate, and the Axial Symptoms of Parkinson Disease

**DOI:** 10.3389/fneur.2018.00806

**Published:** 2018-09-26

**Authors:** Ruth L. O'Gorman Tuura, Christian R. Baumann, Heide Baumann-Vogel

**Affiliations:** ^1^Center for MR Research, University Children's Hospital Zurich, Zurich, Switzerland; ^2^Department of Neurology, University Hospital Zurich, Zurich, Switzerland

**Keywords:** Parkinson disease, magnetic resonance spectroscopy, GABA, glutamate, basal ganglia, prefrontal cortex, gait, axial symptoms

## Abstract

**Introduction:** The axial symptoms of Parkinson disease (PD) include difficulties with balance, posture, speech, swallowing, and locomotion with freezing of gait, as well as axial rigidity. These axial symptoms impact negatively on quality of life for many patients, yet remain poorly understood. Dopaminergic treatments typically have little effect on the axial symptoms of PD, suggesting that disruptions in other neurotransmitter systems beyond the dopamine system may underlie these symptoms. The purpose of the present study was to examine the relationship between the axial symptoms of PD and GABA and glutamate levels quantified with magnetic resonance spectroscopy.

**Methods:** The participant group included 20 patients with PD and 17 healthy control participants. Water-scaled GABA and Glx (glutamate + glutamine) concentrations were derived from GABA-edited MEGA-PRESS spectra acquired from the left basal ganglia and prefrontal cortex, and additional water-scaled Glx concentrations were acquired from standard PRESS spectra acquired from the pons. Spectra were analyzed with LCModel. The axial symptoms of PD were evaluated from subscales of the Unified Parkinson's Disease rating scale (MDS-UPDRS).

**Results:** PD patients demonstrated significantly higher GABA levels in the basal ganglia, which correlated with the degree of gait disturbance. Basal ganglia Glx levels and prefrontal GABA and Glx levels did not differ significantly between patient and control groups, but within the PD group prefrontal Glx levels correlated negatively with difficulties turning in bed. Results from an exploratory subgroup analysis indicate that the associations between GABA, Glx, and axial symptoms scores are typically more prominent in akinetic-rigid patients than in tremor-dominant patients.

**Conclusion:** Alterations in GABAergic and glutamatergic neurotransmission may contribute to some of the axial symptoms of PD.

## Introduction

Parkinson disease (PD) is a progressive neurodegenerative disorder thought to affect over 4 million patients worldwide (1). The core motor symptoms of PD include akinesia, rigidity, resting tremor, and postural and balance difficulties, but patients can also suffer from a broad spectrum of non-motor symptoms (2). While some of the motor symptoms can be improved by dopaminergic (e.g., levodopa) therapy, the non-motor symptoms and the “axial” motor symptoms, including difficulties with gait, posture, speech, swallowing, and postural stability, typically do not respond well to dopaminergic therapy (3). These axial symptoms have a severe, long-lasting negative impact on quality of life and have been identified as a top research priority by patients, family members, and carers affected by PD, second only to the overarching aim to find an effective cure (2).

The neural origin of the axial symptoms of PD is not well-understood, and treatment options are limited. However, converging evidence indicates that disruptions in other neurotransmitter systems beyond the dopamine system are present in PD and may underlie some of the non-motor and axial symptoms (4–6).

Two of the other major neurotransmitter systems implicated in basal ganglia regulation and in PD, namely the glutamate and γ-amino butyric acid (GABA) systems, can be probed non-invasively using magnetic resonance spectroscopy (MRS) (7). Previous MRS studies in PD have reported increased GABA in the pons, basal ganglia, and thalamus (8–10), decreased cortical glutamate (11), and an increased GABA/glutamate ratio in the substantia nigra (12). In other neurodegenerative disorders, altered GABA and glutamate levels have been linked to symptoms which are also present in PD, including cognitive impairment (13, 14) and depression (15). In mice, altered GABA and Glycine neurotransmission was observed to trigger the cardinal features of rapid eye movement sleep behavior disorder (RBD) (16), a premotor symptom of PD which may represent a biomarker for overall disease severity (17). However, few studies have examined altered GABA and glutamate levels in relation to PD symptomatology, and currently the link between alterations in the GABAergic and glutamatergic systems and the axial symptoms of PD remains unclear. The purpose of the present study was to examine the relationship between the axial symptoms of PD, GABA, and glutamate levels in the basal ganglia and prefrontal cortex, and glutamate levels in the pons. Based on previous reports, we hypothesized that patients with PD would show increased GABA levels in the basal ganglia and decreased glutamate in the prefrontal cortex and pons, and that more pronounced abnormalities in the GABA and glutamate levels would correlate positively with the severity of the axial symptoms of PD.

## Materials and methods

### Participants

The patient group consisted of 20 patients with PD (4 female, mean age 63 years, range 50–75) recruited from the Neurology Department of the University Hospital of Zürich, Switzerland (see Table 1 for demographics). All 20 patients had bilateral impairment, and 9/20 patients were treated with levodopa without dopamine agonists, while 11/20 patients were treated with a combination of levodopa and dopamine agonists. Patients were enrolled consecutively. Seventeen healthy control participants (4 female, mean age 62 years, range 28–77) with no history of neurological or psychiatric illness were also recruited. All participants gave written and verbal informed consent to participate in the study, which was approved by the cantonal ethics committee of the Canton of Zürich, Switzerland.

**Table 1:** Patient characteristics and MDS-UPDRS scores.

**Patient**	**Disease duration (years)**	**PD Type**	**Most affected side**	**LED**	**MDS-UPDRS**			
					**I**	**II**	**III**	**IV**
1	18	Akinetic rigid	Right	1500	22	14	31	8
2	14	Tremor-dominant	Right	1300	18	10	41	6
3	5	Tremor-dominant	Right	600	8	15	13	6
4	6	Tremor-dominant	Left	900	17	16	31	4
5	11	Akinetic rigid	Left	1550	15	14	46	9
6	11	Akinetic rigid	Right	1200	10	12	18	1
7	8	Tremor-dominant	Left	800	8	7	25	6
8	8	Akinetic rigid	Left	1500	11	10	27	10
9	16	Akinetic rigid	Right	950	12	14	26	10
10	9	Tremor-dominant	Left	800	13	17	21	8
11	5	Akinetic rigid	Left	750	11	17	26	8
12	11	Akinetic rigid	Left	1500	20	14	31	10
13	12	Akinetic rigid	Right	1550	7	17	33	6
14	8	Tremor-dominant	Left	450	12	14	20	10
15	3	Akinetic rigid	Left	650	23	11	32	0
16	10	Akinetic rigid	Right	850	14	10	21	9
17	12	Akinetic rigid	Right	900	3	5	17	5
18	12	Tremor-dominant	Left	1050	8	12	40	7
19	4	Tremor-dominant	Left	1600	13	15	31	7
20	2	Tremor-dominant	Left	775	15	5	29	6

### MRI/MRS acquisition and analysis

Magnetic resonance imaging and spectroscopy data were acquired with a 3T GE MR750 scanner (GE Healthcare, Milwaukee, WI, USA), using an 8-channel receive-only head coil. On the day of the scan, patients were scanned at a time when their dopaminergic medication was wearing off, before their next dose could be taken. The MRI protocol included a 3D T1-weighted fast spoiled gradient echo (SPGR) scan (echo time (TE) = 3 ms, repetition time (TR) = 8 ms, inversion time (TI) = 600 ms; voxel resolution = 1 × 1 × 1 mm^3^, flip angle = 8°), used for localisation of the MRS voxels and correction of the GABA and glutamate levels for partial volume CSF contamination (18).

**Figure 1:**
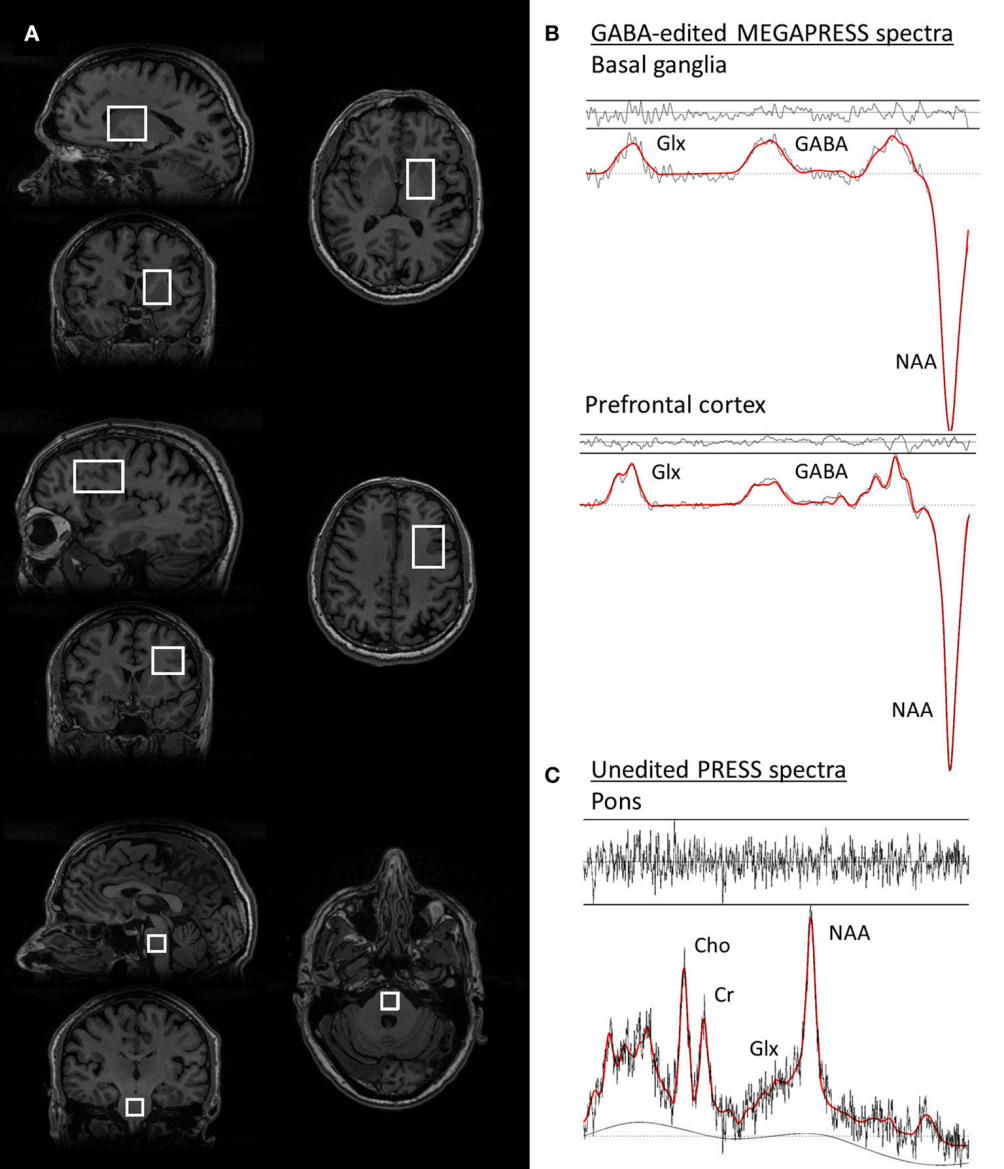
Voxel positions and representative spectra from one PD patient. **(A)** voxel positions from the basal ganglia, prefrontal cortex, and pons voxels, respectively. **(B)** GABA-edited MEGAPRESS spectra from the basal ganglia (top) and prefrontal cortex (middle). The spectral data are plotted in black and the LCModel fit for each spectrum is overlaid in red. **(C)** PRESS spectrum from the pons, with the LCModel fit overlaid in red. For all spectra, the residuals of the fit are plotted above each spectrum.

Single voxel PRESS spectra were acquired from a 3.4 mL (15 × 15 × 15 mm3) voxel in the pons, with TE = 35 ms, TR = 3000 ms, 128 spectral averages, and 16 unsuppressed water reference lines, resulting in a total scan time of 7 min, (see Figure 1 for an illustration of the voxel positions and representative spectra).

Single-voxel GABA-edited spectra were acquired from a 30 mL voxel centered on the left basal ganglia using the MEGAPRESS method (19), with TE = 69 ms, TR = 1800 ms, 320 spectral averages (160 edit ON/OFF pairs), and an eight-step phase cycle. MEGA-editing was achieved with 16-ms Gaussian editing pulses applied at 1.9 and 7.5 ppm in alternate spectral lines. For each metabolite spectrum, 16 unsuppressed water reference lines were also acquired, resulting in a total acquisition time of 10 min. To achieve a consistent voxel position between participants, voxels were prescribed on an axial plane where the putamen was widest in the lateral (right-left) direction, such that the anterior and medial borders of the voxel were aligned with the anterior and medial margins of the head of the caudate nucleus.

Additional GABA-edited MEGAPRESS spectra were collected from a subset of *n* = 31 participants (*n* = 16 PD patients, *n* = 15 controls), using a 30 mL voxel in in the left prefrontal cortex, with TE = 69 ms, TR = 1800 ms, 320 spectral averages (160 edit ON/OFF pairs), and an eight-step phase cycle. MEGA-editing was achieved as described above for the basal ganglia. In order to achieve a consistent voxel position between participants, the voxel was localized according to a standard set of anatomical measurements. (20).

Spectra were analyzed with LCModel version 6.31-H (21). Edited MEGA-PRESS spectra were analyzed with a simulated basis set including basis spectra for GABA, N-acetyl aspartate (NAA), glutamate (Glu), Glutamine (Gln), glutathione, and N-acetyl aspartyl glutamate (NAAG), using the control parameter sptype = mega-press-3 to avoid mis-assignment of the spectral baseline to the GABA peak (22). Since no attempt was made to quantify the co-edited macromolecule underlying the GABA peak in the edited MEGAPRESS spectra, the GABA concentrations should be considered to represent GABA+ rather than pure GABA.

PRESS spectra from the pons were analyzed using a standard GE basis set including basis spectra for Alanine, Aspartate, Creatine, GABA, Glucose, Gln, Glu, glycerophosphorylcholine, phosphorylcholine, lactate, myo-inositol, NAA, NAAG, Scyllo-inositol, and Taurine. For all pontine PRESS spectra the default LCModel baseline fit settings were used.

Metabolite concentrations were calculated in institutional units (I.U.) after referencing each metabolite signal to the unsuppressed water signal and correcting both the metabolite and water signals for partial volume CSF contamination within the voxel (18). Additional water and metabolite relaxation time corrections were not performed. Since our hypotheses focused on alterations in the GABA and glutamate systems, only the GABA and Glx (Glu+Gln) levels from the basal ganglia and prefrontal cortex, and the Glx levels from the pons were entered into the statistical analysis (see below for statistical methods).

### Symptom evaluation

Axial symptoms were evaluated from subscales of the Unified Parkinson's Disease rating scale (MDS-UPDRS) (23) including speech/dysarthria (2.1), swallowing (2.3), turning in bed (2.9), arising from a chair (3.9), freezing of gait (2.13, 3.11), gait (3.10), postural stability (3.12), and posture (3.13). Since several symptom scores pertain to gait disturbance, a summary score for the gait symptoms was derived by summing together the individual scores for items 2.13, 3.10, and 3.11, and an overall summary score of the axial symptoms was also derived by adding together the symptom scores from each of the axial subscales. The UPDRS scores were derived while the patients were in the ON medication state.

### Statistical analysis

GABA and Glx concentrations and symptom scores were tested for normality with a Shapiro-Wilk test. Group comparisons (PD vs. control) were assessed with a two-sided *t*-test for normally-distributed variables and a Mann-Whitney test for non-normally distributed variables. A non-parametric bivariate correlation (Spearman's rho) was used to investigate the association between GABA and Glx levels and axial symptom scores from the MDS-UPDRS. In the event of a significant group difference or correlation emerging for Glx, *post-hoc* tests were performed to ascertain if this difference or association was driven by Glu or Gln. Since the patient group included both tremor-dominant and akinetic-rigid PD patients who are known to demonstrate differences in the progression of axial motor symptoms, (24–26) the relationship between GABA and Glx levels and the overall axial summary score was evaluated in the tremor-dominant and akinetic-rigid subgroups separately, after dichotomising patients according to their motor signs at disease onset (27). In the event of a significant association between the axial summary score and neurotransmitter levels emerging in one or both of the subgroups, additional *post-hoc* correlations with the individual symptom scores were performed in the relevant subgroup(s) in order to ascertain which symptoms were likely to underly the observed association with neurotransmitter levels. In order to investigate the influence of medication on the association between neurotransmitter levels and symptom scores, additional *post-hoc* correlations were also performed for all significant associations between GABA, Glx, and symptom scores after controlling for the levodopa equivalent dose. All statistical analyses were performed with SPSS version 22, with a two-tailed significance threshold of *p* < 0.05. No correction was made for multiple comparisons.

## Results

Representative spectra from the pons, basal ganglia, and prefrontal cortex in one PD participant are shown in Figure 1, together with images depicting the voxel positions.

GABA-edited MEGAPRESS data was not collected from one patient with a lesion in the basal ganglia, and the basal ganglia Glx levels were excluded for one patient in whom an artifact was visible over the Glx doublet in the MEGA-PRESS subtraction spectrum.

Glx levels in the pons and GABA levels in the basal ganglia followed a normal distribution, but GABA and Glx levels in the prefrontal cortex and Glx levels in the basal ganglia were not normally distributed (*p* < 0.05, Shapiro–Wilk test). The axial symptom scores and the corresponding summary score were also not normally distributed (*p* < 0.05, Shapiro–Wilk test).

PD and control groups did not differ in terms of age or gender (*p* > 0.5, Mann–Whitney test, see Table 2). PD patients demonstrated increased GABA in the basal ganglia (*t* = 2.874, *p* = 0.007, 2-tailed *t*-test). After removal of one outlier in the PD group with a GABA level 6 standard deviations away from the mean, GABA levels in the basal ganglia remained significantly higher in the PD group (*t* = 3.009, *p* = 0.005, 2-tailed *t*-test). The corresponding Glx data for this outlier was also removed from further statistical analyses. No significant differences were observed between GABA and Glx levels in the prefrontal cortex or Glx levels in the basal ganglia and pons between the PD and control groups.

**Table 2:** Demographic data and neurotransmitter levels for the PD and control groups.

	**PD**	**Controls**	**PD vs. Control**
Age (Median ± IQR)	62.9 ± 11.5	66.2 ± 12.4	*p* = 0.563
(Mean ± SD)	63.6 ± 6.8	62.5 ± 12.8	
Gender	16 M, 4 F	13 M, 4 F	*p* = 0.798
Left Basal Ganglia GABA^§^	4.61 ± 0.57	3.93 ± 0.75	*p* = 0.005^*^
Left Basal Ganglia Glx	8.27 ± 3.5	6.66 ± 2.0	*p* = 0.069
Left Prefrontal GABA	3.48 ± 0.90	2.96 ± 0.96	*p* = 0.114
Left Prefrontal Glx	8.19 ± 0.98	7.98 ± 0.84	*p* = 0.114
Pons Glx^§^	13.6 ± 4.02	14.2 ± 3.16	*p* = 0.58

§ *Indicates a normally distributed variable, described by Mean ± SD*.

*Data are summarized as median ± inter-quartile range and tested with a Mann-Whitney U test unless indicated otherwise. [MRS levels are given in institutional units (I.U.)]*.

**Figure 2:**
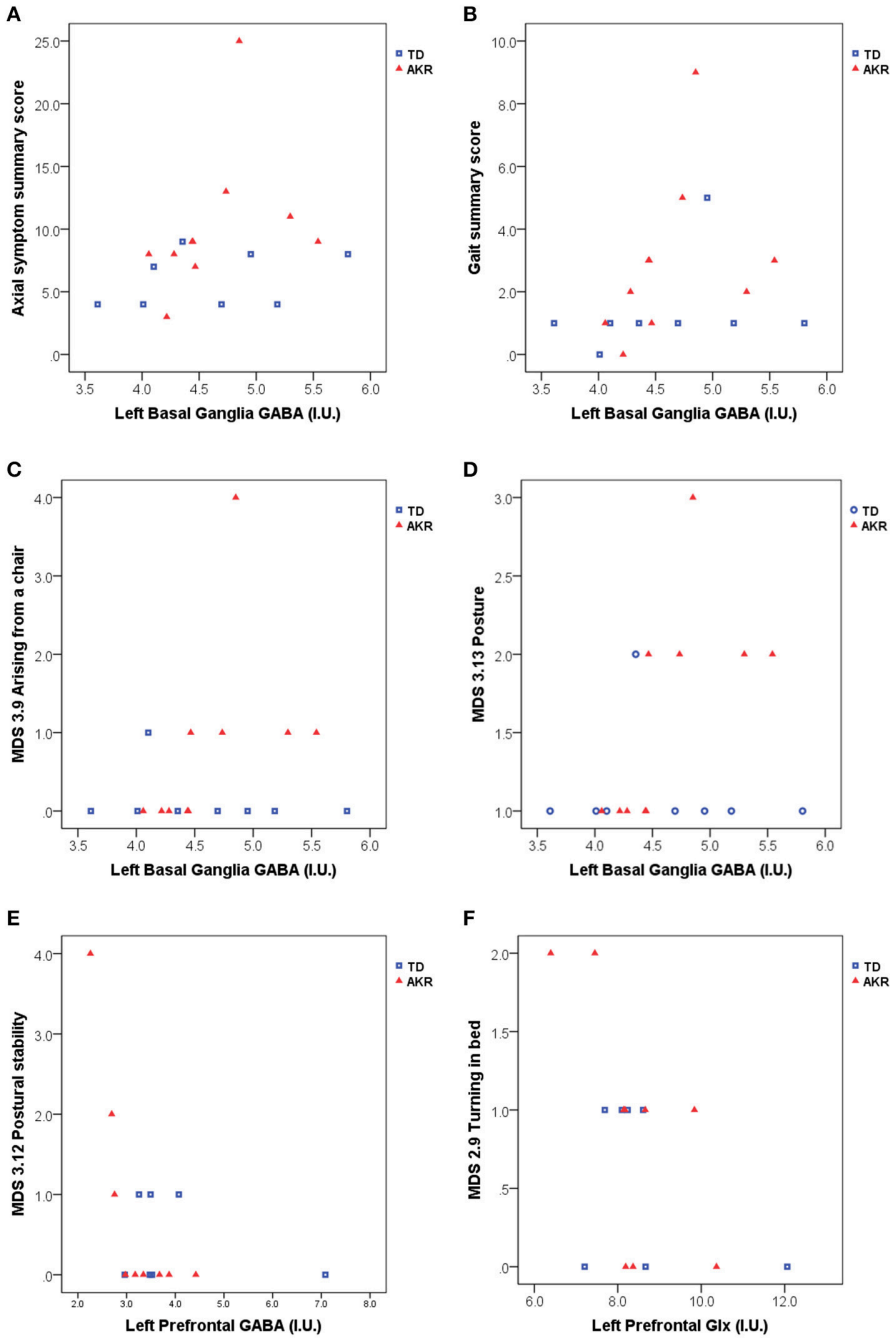
Basal ganglia GABA, prefrontal GABA and prefrontal Glx appear to be associated with axial symptom scores including gait and postural difficulties assessed with the MDS-UPDRS. **(A)** left basal ganglia GABA levels were significantly associated with higher axial symptom summary scores in akinetic-rigid (AKR) patients (*p* = 0.034), but not in tremor-dominant (TD) patients (*p* = 0.403), while the full patient group showed a trend-level association between basal ganglia GABA and axial summary scores (*p* = 0.067). **(B)** Elevated GABA levels were also associated higher gait summary scores (*p* = 0.038, full group). In the AKR subgroup, increased GABA levels were significantly positively associated with difficulties arising from a chair (**C**, *p* = 0.002) and difficulties with posture (**D**, *p* = 0.002). Left prefrontal GABA levels were negatively associated with postural stability in the AKR subgroup (*p* = 0.004, **E**), while left prefrontal Glx levels were negatively associated difficulties turning in bed, both in the full group (*p* = 0.029, **F**), and in the AKR subgroup (*p* = 0.041, **F**).

Significant associations between GABA and Glx concentrations and axial symptom scores are plotted in Figure 2. GABA levels in the left basal ganglia correlated positively with the gait summary score (Spearman's rho = 0.491, *p* = 0.038), as well as the individual gait (MDS 3.10) subscore (Spearman's rho = 0.495, *p* = 0.037) of the motor part of the MDS-UPDRS. Both correlations were present at trend level in the subgroup of akinetic-rigid patients (Gait summary score: Spearman's rho = 0.580, *p* = 0.079, MDS 3.10: Spearman's rho = 0.604, *p* = 0.064), but not in the tremor-dominant subgroup. For the summary score incorporating all the axial symptoms from the UPDRS, basal ganglia GABA levels showed a trend-level association in the full patient group (Spearman's rho = 0.441, *p* = 0.067), which was significant in the subgroup of akinetic rigid patients (Spearman's rho = 0.671, *p* = 0.034), but not in the subgroup of tremor-dominant patients (Spearman's rho = 0.345, *p* = 0.403). In the subgroup of akinetic-rigid patients, basal ganglia GABA levels were significantly correlated with difficulties arising from a chair (MDS 3.9, Spearman's rho = 0.838, p = 0.002 and with posture (MDS 3.13, Spearman's rho = 0.838, *p* = 0.002).

No significant correlations were observed between basal ganglia or pontine Glx levels and the axial symptom scores, but prefrontal Glx levels were negatively correlated with the turning in bed (MDS 2.9) subscore, both in the full patient group (Spearman's rho = −0.546, *p* = 0.029), and in the subgroup of akinetic-rigid patients (Spearman's rho = −0.686, *p* = 0.041). Prefrontal GABA levels were negatively correlated with the postural stability score (MDS 3.12, Spearman's rho = - 0.842, *p* = 0.004), in the akinetic-rigid subgroup only.

The correlations between basal ganglia GABA and the gait subscore and summary score became non-significant after controlling for the levodopa equivalent dose (Spearman's rho = 0.324/0.341, *p* = 0.224/0.196 for the gait summary score and subscore, respectively), as did the correlation between basal ganglia GABA levels and the axial symptom summary score in the akinetic-rigid subgroup (Spearman's rho = 0.403, *p* = 0.322) However, correlations between basal ganglia GABA levels and difficulties arising from a chair (MDS 3.9) remained significant in the akinetic-rigid subgroup after controlling for levodopa equivalent dose (Spearman's rho = 0.845, *p* = 0.008), as did the correlation between basal ganglia GABA and problems with posture (MDS 3.13, Spearman's rho = 0.845, *p* = 0.008). The correlation between prefrontal Glx and turning in bed diminished to trend level after controlling for levodopa equivalent dose (Spearman's rho = −0.491, *p* = 0.074), but the correlation between prefrontal GABA and difficulties with postural stability (MDS 3.12) in the akinetic-rigid subgroup remained significant after controlling for the levodopa equivalent dose (Spearman's rho = −0.76, *p* = 0.047).

## Discussion

Gait and locomotion, turning in bed, arising from a chair, and postural stability all depend on efficient sensorimotor integration (28, 29), which is affected in PD (30–32). The basal ganglia act as an important hub for sensorimotor integration (33, 34) and descending basal ganglia projections to the midbrain have been reported to play an important role in gait and postural control (35, 36). One of the ways in which the basal ganglia are thought to influence sensorimotor integration is by gating or controlling the access of sensory information to motor neurons, via a balance of neurotransmitter activity (30, 37, 38). In PD, the motor systems can become hypo-excitable following an increased inhibition of sensory inputs to the basal ganglia, leading to a diminished motor response to certain sensory stimuli (30). This diminished responsiveness to sensory stimuli is thought to underly the observed difficulties experienced by PD patients in regulating the amplitude of movements in the absence of external visual or auditory cues, when they are dependent on sensory feedback for accurate motor control, (31, 32, 38) and may also contribute to the axial symptoms.

In the present study, the observed link between increased GABA levels in the left basal ganglia in PD patients and the degree of gait disturbance (Figure 2) may be associated with an over-inhibition of the processing of sensory inputs necessary for maintaining posture and initiating locomotion. This observation is consistent with the reported role of GABAergic outputs from the basal ganglia in the control of posture and locomotion (35, 39, 40). However, since locomotion can be initiated by stimulation of the midbrain locomotor region, (36) one would expect corresponding neurotransmitter abnormalities to be observed in the pons, related to problems with gait. In the present study, we were not able to measure GABA in the pons due to the large voxel size required for GABA measurement with MEGA-PRESS, but we did not observe any apparent relationship between the pontine Glx concentrations (from standard PRESS) and problems with gait. However, since the PRESS voxel volume (3.4 mL) is large in comparison to the size of the midbrain locomotor region or the peduculopontine nucleus of the pons, two of the pontine regions implicated in gait control and muscle tone regulation, the lack of an apparent relationship between pontine Glx and posture or gait symptoms may be due to a lack of regional specificity of the pontine MRS measurement. Future studies at higher field strengths (e.g., 7T), where smaller voxel volumes can be used and where GABA can be quantified without the need for spectral editing, may be able to extend the present findings to clarify the link between gait difficulties and pontine neurotransmitter levels.

In the subgroup analysis, the observed link between basal ganglia GABA levels and the gait and axial summary scores seemed to be mostly driven by GABA and symptom changes in the akinetic-rigid subgroup of patients rather than the tremor-dominant patients. Longitudinal studies have shown that the akinetic-rigid subtype of PD presents a risk factor for greater progression of the motor symptoms, including freezing of gait and other axial symptoms. (24, 25, 26) Akinetic-rigid PD patients have also been shown to demonstrate differences in structural and functional connectivity in comparison to tremor-dominant patients, (41, 42, 43) and differences in iron deposition between the subtypes indicates that different pathological mechanisms may underly the observed differences in symptom progression (44). In light of the small group sizes, the subgroup analysis in the present study should be considered exploratory, and we hope these results can be replicated in a larger sample. Given the evidence for differences in brain structure and function as well as symptom progression in different subtypes of PD, it would be interesting to examine differences in the metabolite profile, including neurotransmitter changes, between akinetic-rigid and tremor-dominant PD patients in more detail in a larger cohort.

While basal ganglia GABA levels appeared to be related to gait difficulties, prefrontal Glx levels correlated negatively with difficulties turning in bed (Figure 2), and prefrontal GABA levels correlated negatively with postural stability, in the akinetic-rigid subgroup. The negative correlation between prefrontal Glx and symptom scores would be consistent with previous reports of decreased cortical Glx in PD, (11) under the assumption that patients more severely affected by axial symptoms like turning in bed would have lower prefrontal Glx levels. However, since in the present study no significant differences in prefrontal Glx were observed at the group level, the apparent correlation between frontal Glx and symptom scores seen in the present study cannot be interpreted in the context of abnormal Glx levels in the patient group. The link between prefrontal GABA and posture is also consistent with the putative role of prefrontal and parietal cortical regions in maintaining postural equilibrium, (45) but should also be interpreted with caution in light of the lack of a significant difference in prefrontal GABA between the patient and control groups. It is possible that these associations with individual symptom scores may be affected by outliers, given the small group sizes and the limited distribution of symptoms in some domains. These results should therefore be considered with caution until they can be replicated in a larger sample.

Since the symptom scores were assessed while patients were in the ON medication state, while the MRS was performed just before the next dose was due and thus rather in an OFF state, medication effects could potentially confound the comparison between GABA and Glx levels and the axial symptom scores. While most of the axial symptoms do not typically respond well to levodopa, dopamine replacement therapy has been reported to reduce freezing of gait in patients with PD, (46) and in the present sample the levodopa equivalent dose showed a trend-level association with basal ganglia GABA (*p* = 0.09, Spearman's rho). The effect of levodopa on freezing of gait may explain why the correlations between basal ganglia GABA levels and the gait and axial symptom summary scores diminished in significance after controlling for the levodopa equivalent dose, particularly in akinetic-rigid patients. In contrast, the association between neurotransmitter levels and other symptom scores such as rising from a chair, turning in bed, posture, and postural stability, seemed relatively independent of levodopa effects, but in addition to levodopa 11 patients were additionally medicated with dopamine agonists. Previous studies in children with attention deficit hyperactivity disorder (ADHD) have reported that treatment with dopamine agonists like methylphenidate can reduce striatal Glx levels (47), although methylphenidate did not appear to influence frontal Glx levels, (47) and another study of medication-naïve and medicated adults with ADHD failed to find differences in Glx related to medication status (48). However, since treatment with dopamine agonists or other medications may introduce additional variability into the Glx and GABA signals measured MRS, it would be instructive to clarify the effects of dopaminergic and other medications on the GABA and Glx levels in a future study, where the cohort of PD patients is sufficiently large to enable separation into subgroups according to medication status.

## Conclusion

MRS offers a unique opportunity to study the complex interplay between excitatory and inhibitory neurotransmitter activity both within and outside the motor network. The present pilot study provides evidence of a link between alterations in GABA and glutamate levels and the axial symptoms of Parkinson's disease, lending important insight into the neural origin of these symptoms, and opening up potential avenues for treatment.

## Author contributions

RO: conception, organization, and execution of research project, design and execution of data analysis, and writing of the first draft of the manuscript. CB: conception of Research Project, design and review and critique of data analysis, and review and critique of the manuscript. HB-V: conception, organization, and execution of research project, design and review and critique of data analysis, and review and critique of the manuscript.

### Conflict of interest statement

The authors declare that the research was conducted in the absence of any commercial or financial relationships that could be construed as a potential conflict of interest.
